# Diet and obesity effects on cue-driven food-seeking: insights from studies of Pavlovian-instrumental transfer in rodents and humans

**DOI:** 10.3389/fnbeh.2023.1199887

**Published:** 2023-06-22

**Authors:** Joanne M. Gladding, Laura A. Bradfield, Michael D. Kendig

**Affiliations:** Brain and Behaviour Group, Department of Medical Science, School of Life Sciences, University of Technology Sydney, Sydney, NSW, Australia

**Keywords:** obesity, diet, cues, Pavlovian-instrumental transfer (PIT), food-seeking

## Abstract

Our modern environment is said to be obesogenic, promoting the consumption of calorically dense foods and reducing energy expenditure. One factor thought to drive excess energy intake is the abundance of cues signaling the availability of highly palatable foods. Indeed, these cues exert powerful influences over food-related decision-making. Although obesity is associated with changes to several cognitive domains, the specific role of cues in producing this shift and on decision-making more generally, remains poorly understood. Here we review the literature examining how obesity and palatable diets affect the ability of Pavlovian cues to influence instrumental food-seeking behaviors by examining rodent and human studies incorporating Pavlovian-instrumental transfer (PIT) protocols. There are two types of PIT: (a) general PIT that tests whether cues can energize actions elicited in the pursuit of food generally, and (b) specific PIT which tests whether cues can elicit an action that earns a specific food outcome when faced with a choice. Both types of PIT have been shown to be vulnerable to alterations as a result of changes to diet and obesity. However, effects appear to be driven less by increases in body fat and more by palatable diet exposure *per se*. We discuss the limitations and implications of the current findings. The challenges for future research are to uncover the mechanisms underlying these alterations to PIT, which appear unrelated to excess weight itself, and to better model the complex determinants of food choice in humans.

## 1. Introduction

Obesity and high-fat/high-sugar (HFHS) diets are each associated with impairments to cognition, executive function, and memory (Francis and Stevenson, 2013; O’Brien et al., 2017). These associations are correlative, however, and it is unclear whether obesity is a *cause* of impaired cognition, or whether cognitive impairments and obesity are related because they are both *consequences* of consuming a HFHS diet. This is important to determine for two reasons. First, individuals with overweight and obesity may or may not consume HFHS diets. It is therefore important to determine whether obesity alone is a risk factor for cognitive impairment. Second, there are many individuals of “healthy” weight that do consume HFHS diets, and it is important to determine the risks for this population to their cognitive health.

Therefore, in this focused review we examine how food-seeking behavior is altered by excess weight or the consumption of HFHS diets *per se*, focusing on human and rodent studies using a laboratory procedure designed to study the effects of cues on food-seeking action performance and selection, known as Pavlovian-instrumental transfer (PIT). Preliminary evidence in this emerging field suggests that HFHS diet exposure can directly impair PIT, whereas changes in body weight are neither necessary nor sufficient for impairments. Notably, PIT impairments have been associated with prospective weight gain, and have been observed in populations of rodents predisposed to obesity in the absence of any diet manipulation. We discuss the implications of these findings for understanding the causal relationships between diet, cognition, and weight gain.

### 1.1. Pavlovian-instrumental transfer

**FIGURE 1:**
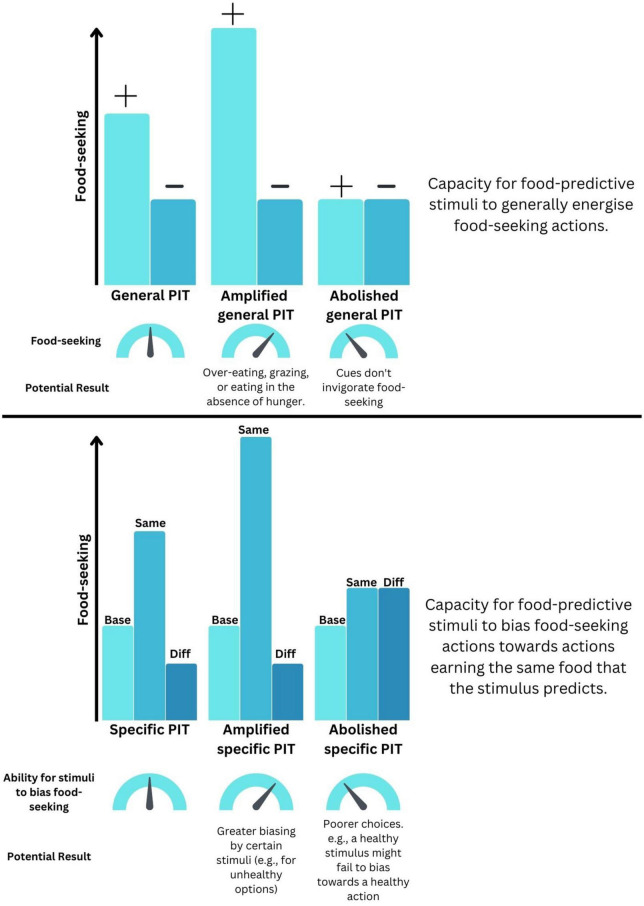
How distortions to Pavlovian-instrumental transfer (PIT) could drive maladaptive food-seeking. **(Top)** Schematic representation of how changes to the ability for predictive cues to invigorate general food-seeking could be distorted. (+) indicates a food-predictive cue whereas (–) indicates a neutral cue. **(Bottom)** Schematic representation of how changes to the ability for predictive cue to bias the selection of foods could become maladaptive. “Base” indicates baseline food-seeking in the absence of predictive cues. “Same” indicates food-seeking for an outcome that is also predicted by that cue. “Diff” indicates food-seeking for an outcome that is different to that predicted by that cue.

During the first Pavlovian stage of PIT, subjects are exposed to cues that predict food. For example, in a human version of the task, different colored lights within a computer-simulated image of a vending machine indicate that the participant will receive various food outcomes such as M&M’s, potato chips, or juice. In the rodent version, a loud noise or tone is paired with the delivery of a grain pellet or a sucrose solution. After several training sessions, an instrumental phase begins in which subjects perform an action to earn food outcomes; for example, pressing a left or right button on the vending machine to earn M&M’s and chips, respectively, whereas rodents learn to press left and right levers to earn pellets and sucrose solution. In the final test phase, known as “transfer,” the subject is presented with the cues and their subsequent action selection is measured. In the general version of the task (general PIT; gPIT), subjects will increase the performance of any or all food-seeking actions during presentation of the food-paired cue relative to a neutral, non-predictive cue or when the cues are absent (top panel of Figure 1). In the human example used here, this would comprise presentations of a juice cue elevating overall responding on both left and right buttons, even though they previously predicted M&M’s and chips. In the specific version (specific PIT; sPIT), the subject will increase performance of the action that produced the same outcome as the cue that is currently being presented. For instance, the cue for M&M’s should elicit pressing on the button that earned M&M’s, and the potato chip cue should elicit pressing on the potato chip button (i.e., Same > Different, bottom panel of Figure 1).

**FIGURE 2:**
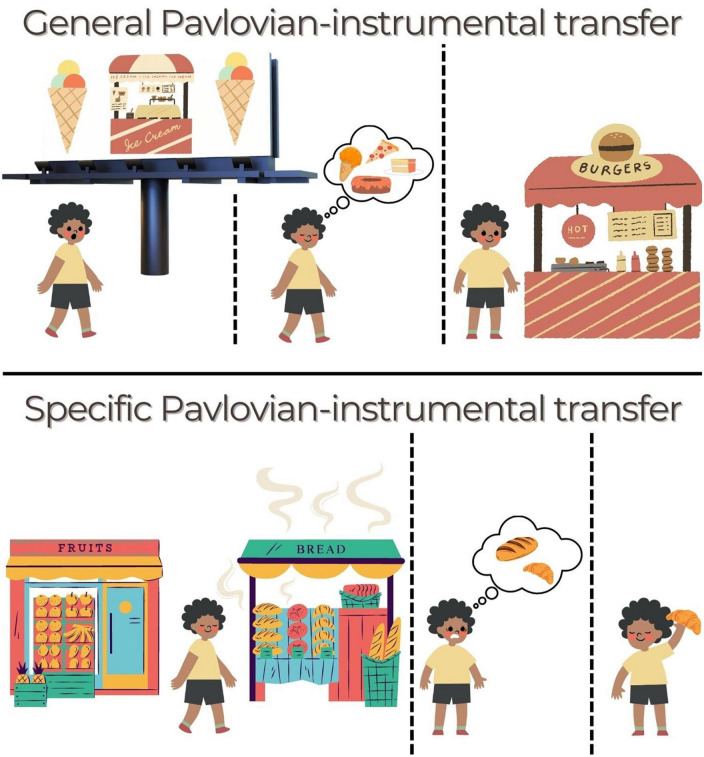
Ways that PIT might operate in daily life to drive food-seeking. **(Top)** General PIT. Someone is walking when they see a billboard for their favorite ice-cream. They continue walking, feeling a little hungry now, before deciding to stop at a burger shop that they see. The billboard acted as a cue that aroused general motivation to seek out food (action), even though the burger (outcome) was not specifically linked to that cue. An amplification of this effect could lead to overeating or indiscriminate grazing. **(Bottom)** Specific PIT. The next morning, they want to get something for breakfast on their way to work but they haven’t decided what. At the end of the street is a bakery and a fruit grocer. As the person approaches, they smell the aromas of freshly baked bread wafting toward them (cue). This guides them to select (action) a fresh croissant (“Same” outcome) rather than a fruit salad. A distortion of this effect could lead to greater biasing by certain cues (e.g., unhealthy foods) or failure to use a healthy cue to select a healthy option.

Figure 2 outlines how the processes measured in general and specific PIT might influence food-seeking behaviors in the real-world. As can be seen from the examples, PIT is a translationally valid measure of the influence of many types of environmental cues on instrumental behavior (Cartoni et al., 2016). Moreover, each type of PIT measures different facets of food-seeking behaviors. For instance, gPIT reflects the motivational regulation of food-seeking by assessing how food-predictive cues invigorate food-seeking behaviors in a non-specific fashion. In other words as depicted in Figure 2, the predictive cue acts to increase general motivational arousal (Corbit and Balleine, 2005). Outcome value is critical for the expression of gPIT: when outcomes are devalued, the predictive cue loses the capacity to invigorate actions (Balleine, 1994; Corbit et al., 2007; Lingawi et al., 2022). In contrast, sPIT reflects the cognitive control of food-seeking by food-predictive cues. Thus, unlike gPIT, in sPIT the retrieval of the sensory-specific properties of the outcome guides behavior to a greater extent than the general motivational drive to eat (Cartoni et al., 2016). Unlike gPIT, expression of sPIT has been argued to be insensitive to changes in outcome value (Corbit et al., 2007; Lingawi et al., 2022; Sommer et al., 2022), although this conclusion has not been unanimously supported (Panayi and Killcross, 2022).

By measuring distinct aspects of cue-guided food-seeking, gPIT and sPIT represent useful tools for examining the effects of obesity and HFHS diet exposure in controlled settings. Of course, this high degree of experimenter control does come with a trade-off, in the sense that learning in the real world is unlikely to be as neatly partitioned as it is in the laboratory (with separate stages of learning for Pavlovian and instrumental phases). However, whilst keeping this caveat in mind, there are several ways in which a deviation from “normal” PIT performance could indicate the presence of maladaptive behaviors that contribute to excess energy intake (see Figure 1). For instance, an amplified gPIT response – wherein a food-predictive cue invigorates food-seeking in a non-specific fashion – could result in overeating or grazing. By contrast, an amplification of sPIT, wherein a food-predictive cue invigorates behavior targeting a specific food – could become problematic if cues signaling unhealthy foods are more prevalent or more strongly bias our responding toward selecting those foods. On the other hand, an abolishment of sPIT and the loss of the capacity to use cues to guide appropriate choice between actions might manifest as “poorer” food choices, if exposure to cues paired with healthy foods no longer leads to selection of that outcome.

## 2. Effects of diet and obesity on general PIT

Increased food-seeking, particularly when food is not required, could contribute to overeating and weight gain over the long-term. Two recent studies have observed that gPIT is amplified in obese-prone relative to obese-resistant rats, wherein a food-predictive cue, relative to a neutral cue, increased instrumental performance for an action that earned another food (Derman and Ferrario, 2018, 2020). Critically, rats used in these studies were maintained on standard chow, with no differences in body weight between obese-prone and obese-resistant strains, since the phenotype is contingent on obesogenic diet exposure (Levin et al., 1997). Thus, these studies suggest that an amplified gPIT response can occur independent of weight or adiposity changes. Of course, it is not clear from this study whether alterations in diet might alter gPIT performance either. Fortunately, however, others have explicitly studied the effects of HFHS diets on gPIT.

A recent study found that gPIT abnormally persisted in mice previously fed a high-fat diet relative to a chow-fed control group when they were tested under conditions of satiety (Gladding et al., 2023). Specifically, mice were fed a chow or high-fat diet for 8 weeks followed by a standard chow diet for 5 weeks, which ameliorated diet-induced weight gain such that body weight did not differ between groups when Pavlovian and instrumental training commenced. The persistence of gPIT in the high-fat diet fed mice despite a change in motivational state indicates a long-term modification in food-seeking, independent of excess body weight and adiposity. In everyday life, humans are exposed to food-predictive cues across the full spectrum of hunger to satiety. If the results of Gladding et al. (2023) are translatable, it could suggest that when the ability for motivational state to control cue-driven food-seeking is lost, eating in the absence of hunger could increase and subsequently promote weight gain. It will be important for future studies to further replicate and elucidate these effects, but this is not without its challenges. Diet-induced obesity alters Pavlovian conditioning and the motivation to perform instrumental actions in rodents (Harb and Almeida, 2014; Tracy et al., 2015; Shipman and Corbit, 2022). As such, experiments will need to be designed carefully to avoid differences in baseline performance confounding any subsequent differences in PIT. Altogether, there is evidence that alterations to gPIT can arise prior to the development of obesity and after the consumption of an obesogenic diet. Yet, both have been observed in the absence of excess adiposity.

Despite the potential ecological validity of gPIT there have been few clinical investigations of it. To our knowledge, there are only two human studies have observed gPIT in participants of normal-weight (Watson et al., 2014; Degni et al., 2022). In these studies, where participants completed a computerized task, food-predictive cues biased choice toward the signaled food image (sPIT; Watson et al., 2014) while also enhancing the general vigor of instrumental responding (gPIT; Watson et al., 2014; Degni et al., 2022). In addition, there is one study that compared gPIT performance in participants with normal weight or obesity. Unfortunately, the effect was not elicited in either group, despite the experimenter’s using real juice outcomes and not food images (Meemken and Horstmann, 2019). Participants’ hunger ratings were low though (3.6 out of 10) indicating that the test was conducted under relative levels of satiety which may have abolished gPIT.

Overall, there is sparse yet emerging evidence regarding how gPIT might coincide with obesity. To advance our understanding, future investigations should explore the effects of the types of food used as outcomes (e.g., low versus high calorie), parameters of the conditioning paradigm (e.g., food images versus real outcomes), and cues (visual, auditory, or olfactory), as these factors all have implications for the generalizability of results to real-world settings. Given that gPIT is sensitive to manipulations of outcome value, particular attention should be given to food palatability and desirability in these studies.

## 3. Effects of diet and obesity on specific PIT

Because sPIT involves the choice between multiple actions that earn multiple outcomes, it is possible to envisage multiple ways in which the cognitive processes that underlie it could be altered by dietary changes and/or obesity. For instance, alterations to sPIT could manifest as the inability to choose a healthy food option that is advertised, or as an exaggerated response (in terms of consumption or choice) in the presence of adverts for unhealthy options. Alternatively, it is possible that whereas a food cue might elicit a specific action in one individual, it is possible that it could generate a more general food-seeking response in another individual, who then seeks multiple types of food. The few studies that have studied the effects of obesity and/or diet on sPIT shed some light onto these processes.

A study in outbred rats showed that the magnitude of sPIT at baseline negatively correlated with subsequent weight gain post-5 weeks of junk-food consumption. That is, a weaker expression of sPIT predicted greater weight gain later (Derman and Ferrario, 2020). Accordingly, sPIT was abolished in obese-prone rats (Derman and Ferrario, 2020) and in rats fed a junk-food diet for 6 weeks (Kosheleff et al., 2018). Notably, although the latter junk-food food fed rats could not use cues to guide their choice between actions, their body weight did not differ significantly from their chow-fed counterparts. Together, these studies support the notion that diet (and/or predisposition to obesity) was abolishing the selectivity of the food-seeking response. In contrast, in another recent study mice fed a high-fat diet for 8 weeks did not show any impairments in sPIT (Gladding et al., 2023). However, these mice were returned to a chow diet for 5 weeks prior to behavioral testing, and it is possible that this diet switch ameliorated the effects of the high-fat diet on choice behavior. Indeed, several studies have observed improvements in various cognitive functions in rodents switched from HFHS diets to regular chow (Sobesky et al., 2014; Boitard et al., 2016; Tran and Westbrook, 2017; Kendig et al., 2018; McLean et al., 2018; Altherr et al., 2021). Nevertheless, these few studies indicate that it is not excess weight *per se* that drove the changes in cue-driven choice.

In humans, three studies have investigated the link between obesity and sPIT, with mixed results. Watson et al. (2017) found that for individuals with obesity, choice between actions was more heavily biased by cues that predicted palatable or high-calorie options than it was for normal weight individuals. However, another study found that while overweight participants showed a stronger sPIT effect than healthy-weight controls, participants with obesity did not differ from either group (Lehner et al., 2017). The third study likewise found no differences in choice behavior between individuals with obesity versus individuals of healthy-weight (Meemken and Horstmann, 2019). Thus, while there is evidence for sPIT being abolished (Kosheleff et al., 2018; Derman and Ferrario, 2020), enhanced (Lehner et al., 2017; Watson et al., 2017), or left intact (Meemken and Horstmann, 2019; Gladding et al., 2023) across different obesity models, the link to excess weight is weak. Moreover, it is not possible from these studies to disentangle the effects of weight gain itself and the dietary choices that might have caused the weight gain. Together, therefore, these studies call into question the idea that obesity induces cognitive deficits directly, and instead suggests any such relationship is correlational and potentially linked to consumption of an obesogenic diet.

## 4. Discussion

Although obesity is associated with changes across several cognitive domains, the idea that obesity is characterized by exaggerated responses to food cues or food itself has been challenged by recent data (Hagan et al., 2020; Morys et al., 2020; Wall et al., 2020). Instead, there is emerging evidence that exposure to HFHS diets exert a larger effect on food-seeking behavior than obesity. Studies using gPIT show that food-predictive cues energize general food-seeking behavior in obese-prone, relative to obese-resistant, rats prior to differences in weight gain (Derman and Ferrario, 2018, 2020), and this energizing effect persists in non-obese mice previously fed a high-fat diet (Gladding et al., 2023). Similarly, studies utilizing sPIT show that choice between actions is altered in obese-prone rats (Derman and Ferrario, 2020), non-obese junk-food fed rats (Kosheleff et al., 2018), and individuals that are overweight (Lehner et al., 2017) or, in some cases, obese (Watson et al., 2017). Collectively, consuming a HFHS diet, predisposition to weight gain, and increased body mass index (BMI) have all been associated with greater susceptibility to food-related cues, though in our view the relationship to the latter factor is correlational more than causal. Thus, at least based on the studies reviewed here, it appears that HFHS diets are the more likely cause of cognitive deficits, in regard to responses to food cues, than is weight gain or obesity.

There is, however, a complexity to the evidence reviewed here that we have not yet acknowledged. That is, there appears to be a bidirectional relationship between HFHS diet intake and cognitive impairment as measured by PIT tasks in a manner that is similar to that posited by the “vicious cycle” model of obesity and cognitive impairment, which focuses on hippocampal function (Hargrave et al., 2016). This account hypothesizes that HFHS diet-induced impairments to hippocampal function reduce the capacity for internal satiety signals to suppress appetitive behaviors in the presence of food-predictive cues. Consequently, food-predictive cues are better able to elicit consumption in subjects on a HFHS diet, promoting excess energy intake and weight gain in a continuing cycle. The model focuses on the loss of sensitivity to *internal* satiety cues produced by HFHS diet consumption (i.e., interoceptive functions mediated by the hippocampus) but acknowledges that increasing exposure to, or salience of, *external* cues (i.e., exposure to food advertisements in day-to-day life) may also perpetuate the cycle.

The studies of PIT reviewed here are consistent with this model in showing that HFHS diets can alter cue-driven food-seeking behavior, but that differences in PIT are evident in rodents predisposed to obesity and predict weight gain in rodents fed HFHS diets. This presents a challenge for studying the effects of HFHS diets on food-related tasks in humans: if HFHS intake is associated with impairments in food-seeking behavior, does this reflect a true dietary effect or a pre-existing trait difference that has influenced dietary habits? Indeed, insults other than HFHS diet consumption (e.g., brain injury) might impair aspects of cognition that subsequently drive HFHS intake to initiate the cycle (Hargrave et al., 2016). This quandary highlights the importance of identifying the underlying cognitive or sensory processes that lead to impairments in PIT. One obvious candidate is cue reactivity, which one meta-analysis found to be associated with aspects of eating behavior and weight gain (Boswell and Kober, 2016). Assessing PIT (or other tasks) both pre- and post-HFHS diet exposure might also improve the ability to tease apart the direction of causal effects. Because this is so difficult to do in humans in a highly controlled manner, this further highlights the importance of continued animal studies in this area. Moreover, because humans do not necessarily eat only a diet of junk-food, as rodents in the laboratory often do, to enhance ecological validity animal studies should continue to investigate how the pattern of HFHS diet access (e.g., acute, intermittent, chronic) affects cue-driven food-seeking behavior and the learning of cue-outcome relationships with various contingencies.

### 4.1. Neurobiological bases of dietary effects on PIT

It is worth briefly discussing the neural changes that may underpin dietary effects on PIT. There is a wealth of evidence that HFHS diets alter reward-related brain circuitry, yet direct links to PIT expression have yet to be determined. Lesion, inactivation and imaging studies in rodents and humans indicate that the central nucleus of the amygdala and the core subregion of the nucleus accumbens are required for gPIT, whereas the basolateral nuclei of the amygdala and nucleus accumbens shell subregion are necessary for sPIT (for a comprehensive review of brain regions involved in PIT, see Cartoni et al., 2016). Notably, HFHS diets have been shown to produce inflammation, insulin resistance, glutamatergic dysfunction, and dopaminergic dysfunction across these brain regions, providing candidate mechanisms for the behavioral deficits in PIT that HFHS diets can produce.

In the amygdala, few studies have compared diet-induced inflammatory changes in the central and basolateral nuclei. One such study in rats found that high-fat diet induced increases in markers of microglial function and morphology were more pronounced in the central rather than basolateral amygdala (Spencer et al., 2019). Another study also reported increased markers of inflammation in the central amygdala following high-fat diet exposure (Castro et al., 2013), but did not investigate the basolateral amygdala. Rodent studies looking at whole amygdala have produced additional evidence of dietary-induced neuroinflammation, albeit with a somewhat mixed profile of expression. Exposure to a diet high in fat/or sugar increased levels of the pro-inflammatory cytokine TNFα but not IL-6 or IL-10 in rats (Reis et al., 2023), whereas it increased TNFα and not IL-1β in mice (Akter et al., 2020). In another study, however, the IL-6 and TNFα pro-inflammatory cytokines were both elevated in rats following a high-fat diet (Noronha et al., 2019). Altogether, there is plethora of evidence that inflammation occurs throughout the amygdala following exposure to HFHS diets.

Other studies have reported neuroinflammatory and neuroplastic changes in the nucleus accumbens after HFHS diet exposure. For instance, 6 weeks of HFHS cafeteria diet treatment in mice increased microglial activation and levels of IL-1β and IFN-γ in the core and shell subregions (Gutiérrez-Martos et al., 2018). This was associated with deleterious morphological changes in neuronal spine density and plasticity (Gutiérrez-Martos et al., 2018). Similarly, another study in mice showed that 12 weeks of high-fat diet treatment induced plasticity-related changes in the nucleus accumbens, as indicated by increased BDNF and ΔFosB levels (Sharma and Fulton, 2013). Such diet-induced changes in both the amygdala and nucleus accumbens have been linked to increases in anxiolytic (Noronha et al., 2019; Akter et al., 2020; Reis et al., 2023) and depressive-like (Sharma and Fulton, 2013) behaviors. Importantly, for the current review, direct and indirect projections exist between the nucleus accumbens subregions and nuclei of the amygdala (Heimer et al., 1991; Park et al., 2019) which are critical for PIT (Corbit and Balleine, 2005; 2011; Corbit et al., 2016). Therefore, although links to aberrant PIT and other forms of decision-making have not been interrogated directly, diet-induced changes in these regions is a plausible candidate mechanism for the discussed alterations in PIT expression.

Wallace and Fordahl (2022) proposed one potential mechanism for how HFHS diets could alter cognition. Specifically, they suggested that HFHS consumption promotes neuroinflammation, leading to decreased dopamine synthesis, release, and reuptake in reward-related brain regions. Because nucleus accumbens dopamine activity normally correlates with, and is necessary and sufficient for, PIT (Lex and Hauber, 2008; Peciña and Berridge, 2013; Collins et al., 2016), it is possible that such a dysfunction in dopamine could mediate the dietary effects on PIT. There are several lines of evidence that are consistent with such an account. For instance, there is evidence that glutamate release from the basolateral amygdala targets medium spiny neurons in the nucleus accumbens shell to promote sPIT, and that blocking dopamine receptor D1 (DRD1) receptors on these neurons is sufficient to abolish sPIT (Laurent et al., 2014). It is also consistent with findings showing that high-fat diet consumption reduces DRD1 and dopamine receptor D2 (DRD2) receptor expression in the nucleus accumbens in mice (Carlin et al., 2016). Finally, although 6 weeks of high-fat diet consumption impaired insulin function and dopamine reuptake in the nucleus accumbens of mice, pharmacologically restoring insulin signaling at dopamine terminals restored dopamine reuptake (Fordahl and Jones, 2017). Interestingly, the precise role of DRD2 receptors in PIT is currently unknown but the functional consequences of decreased signaling at these receptors include compulsive food intake, hyperphagia, and compensatory overeating (Johnson and Kenny, 2010).

Overall, the literature shows that HFHS diets induce a broad range of neurobiological alterations, including insulin resistance, dopamine dysfunction, and neuroinflammation. Based on the available evidence we suggest that it is the diet-induced alterations to accumbal dopamine receptor expression (which rely on inputs from the amygdala) that could underlie diet-induced changes to PIT. A priority for future work is to produce direct causal evidence for this hypothesis, as well as identifying the specific mechanisms of other diet-induced alterations in cognition.

### 4.2. Challenges in using PIT to study diet and obesity

While the rodent evidence suggests that diet composition is a key determinant of changes to food-seeking behavior, the effects of diet on food-seeking have not been well explored in human studies. This could be achieved by incorporating measures of habitual diet and/or acute food intake manipulations in studies of PIT and related behaviors. These options are not always practical, however, and measuring energy intake may be prone to human error.

Careful consideration should be given to methods of assessing obesity in future studies. The human PIT studies discussed here all categorized participants by BMI. Yet, obesity is a heterogenous condition and there is high variability within behavioral datasets when participants are categorized exclusively by BMI. Indeed, not all people with an elevated BMI are metabolically “unhealthy” (Rask-Andersen and Johansson, 2023) and people of normal weight can develop the metabolic syndrome (Stefan et al., 2017). While BMI is a quick and practical option with established norms, it is a poor indicator of adiposity and does not consider the distribution of body fat, which is relevant for several pathologies (Fox et al., 2007; Nuttall, 2015; Rask-Andersen and Johansson, 2023). Because evidence from the rodent literature indicates that altered sensitivity to PIT is more likely a moderator that drives overconsumption and weight gain, rather than a consequence of it (Derman and Ferrario, 2018, 2020; Kosheleff et al., 2018), identifying a link between obesity and PIT calls for a finer resolution of the prognostic factors – beyond BMI. Accordingly, the role of individual differences in neurobiology and reward motivation on responsivity to food-related cues and subsequent vulnerability to weight gain was recently outlined (Ferrario, 2020). Future studies could endeavor to directly assess body fat mass, abdominal adiposity, and other metabolic variables as opposed to BMI alone (Davillas and Benzeval, 2016).

Methodological differences between PIT studies are also important to consider, including the palatability and energy density of food rewards used. For example, studies have used high versus low calorie foods (Watson et al., 2017), palatable and highly caloric pellets (Kosheleff et al., 2018; Derman and Ferrario, 2020), snacks selected by participants (Lehner et al., 2017), and equally palatable juices (Meemken and Horstmann, 2019) as rewards. These options provide good ecological validity in understanding food-seeking and choice, as in day-to-day life people might face choices between two similarly palatable options or choices where a more preferred, palatable option is available. On the other hand, the different food types used across studies might explain their incongruent results. Even though the hedonic properties of food should not be essential for intact choice during sPIT (Corbit et al., 2007; Lingawi et al., 2022; Sommer et al., 2022), they could still alter the strength of sPIT expression (Corbit et al., 2007; Watson et al., 2014).

Finally, the use of behavioral tests involving food rewards should be employed carefully when studying HFHS diets, which can alter the motivational value and palatability of the rewards used in conditioning (Harb and Almeida, 2014; Tracy et al., 2015). A recent 8-week trial of daily HFHS snack intake presented an elegant solution by assessing associative learning in a paradigm that paired neutral auditory and visual cues instead of food-paired stimuli or food rewards (Thanarajah et al., 2023). fMRI analyses taken during the neutral associative learning task revealed that daily consumption of a HFHS snack elevated neural responses in brain regions involved in cue-outcome learning. This indicates that unhealthy diets can indeed incur neural adaptations that might impact how we form sensory associations, which is of course relevant in the context of food-paired stimuli and rewards. Refining the clinical methodologies and reproducing previous findings is necessary to gain insight into the power that cues have over food-seeking, whether it depends on food type, and how this might be altered in the development of obesity.

### 4.3. Conclusion

There has been significant research into the effects of obesogenic diets on higher-order cognitive functions and memory, yet there is considerable need for greater exploration and replication of the effects of these diets on food-seeking behaviors. This is because changes to these behaviors might perpetuate a cycle of unhealthy dietary choices and health problems. As reviewed here, the evidence so far indicates that predisposition to metabolic disturbance and the consumption of HFHS diets dysregulates the ability to use food-predictive cues to moderate food-seeking actions. There is, however, no clear link between excess body weight and changes to cue-driven food-seeking. Together, these studies suggest that obesity and/or weight gain is not necessary, but HFHS diets are sufficient, for food-cue related cognitive deficits. In other words, the relationship between overweight and obesity and cognitive impairment appears correlational, with an unhealthy diet perhaps the common cause. This is difficult to parse out in human studies when BMI is used to categorize participants without more detailed measurement of dietary patterns. Therefore, future research in both rodents and humans will help to continue to clarify the causal effects of obesogenic diets on cue-driven food-seeking behaviors such as PIT and the mechanisms that underlie them.

## Author contributions

JG conceptualized the manuscript with support from LB and MK. All authors wrote the manuscript and read and approved the final manuscript.
